# 

**Published:** 1904-02-15

**Authors:** 


### CONTENTS.

Some Facts and Phases of Syphilis that Interest the Dentist, *By Don M. Graham, M.D., D.D.S.* 55

What to Do with Porcelain, *By W. T. Reeves, D.D.S.* 60

Nitrous Oxid Anesthesia, - *By Dr. F. M. Richardson* 68

Continuous Local Infection, *By Rickman J. Godlee, M.S.* 74

Some Advantages of a Swaged Shell Crown, *By W. A. Knowles, M.D., D.D.S.* 82

Hot Water as an Obtundent, *By S. H. Guilford, D.D.S., PH.D.* 87

The Value of a Correct Articulation in Prosthodontia, *By Harvey M. Kirk, D.D.S.* 90

An Improved Method of Making Crowns, *By Herbert Ziele* 95

Editorial, - - - - - - - - - - 98

Announcements, - - - - - - - - 99

Bibliography, - - - - - - - - 100

Obituary, - - - - - - - - - 101

Indents, - - - - - - - - - 102

